# Pelvic Girdle Reconstruction Based on Spinal Fusion and Ischial Screw Fixation in a Case of Aneurysmal Bone Cyst

**DOI:** 10.1080/13577140310001644805

**Published:** 2003

**Authors:** Matthias Honl, Florian Westphal, Volker Carrero, Michael Morlock, Karsten Schwieger, Ekkehard Hille, G. Delling

**Affiliations:** 1 Department of Orthopaedic Surgery Eilbek General Hospital Friedrichsberger Str. 60 Eilbek Hamburg 22081 Germany; 2 Biomechanics Section Technical University Hamburg-Harburg Hamburg-Harburg Germany; 3 Charité University Hospital Berlin Germany; 4 Department of Bone Pathology University Hamburg Hamburg Germany

## Abstract

A case of lytic lesion of the pelvis in a 23-year-old woman is presented. A biopsy led to the diagnosis aneurysmal bone cyst (ABC). Due to the histologically very aggressive growth of the tumor, a low malignant osteosarcoma could not be excluded. In an initial operation the tumour, affecting the sacrum, the iliac crest and the lower lumbar spine was resected. Temporary restabilisation of the pelvic ring was achieved by a titanium plate. The histological examination of the entire tumour confirmed the diagnosis ABC. After 6 months, the MRI showed no recurrence. The observed tilt of the spine to the operated side on the sacral base prompted a second surgical procedure: a transpedicular fixation of L5 and L4 was connected via bent titanium stems to the ischium, where the fixation was achieved by two screws. This construction allowed the correction of the base angle and yielded a stable closure of the pelvic ring. The patient has now been followed for 6 years: the bone grafts have been incorporated and, in spite of radiological signs of screw loosening in the ischium, the patient is fully rehabilitated and free of symptoms. Pedicle screws in the lower spine can be recommended for fixation of a pelvic ring discontinuity.


*Sarcoma*, September/December 2003, *VOL. 7, NO. 3/4, 177–182*

Taylor & Francis healthsciences

CASE REPORT

### Pelvic girdle reconstruction based on spinal fusion and ischial screw fixation in a case of aneurysmal bone cyst

MATTHIAS HONL[1], FLORIAN WESTPHAL[1], VOLKER CARRERO[3], MICHAEL MORLOCK[2], KARSTEN SCHWIEGER[1], EKKEHARD HILLE[1] & G. DELLING[4]

*[1]Department of Orthopaedic Surgery, General Hospital Eilbek, Eilbek, Germany, [2]Biomechanics Section, Technical University Hamburg-Harburg, Hamburg-Harburg, Germany, [3]Charité University Hospital, Berlin, Germany, and [4]Department of Bone Pathology, University Hamburg, Hamburg, Germany*

**Abstract**

A case of lytic lesion of the pelvis in a 23-year-old woman is presented. A biopsy led to the diagnosis aneurysmal bone cyst (ABC). Due to the histologically very aggressive growth of the tumor, a low malignant osteosarcoma could not be excluded. In an initial operation the tumour, affecting the sacrum, the iliac crest and the lower lumbar spine was resected. Temporary restabilisation of the pelvic ring was achieved by a titanium plate. The histological examination of the entire tumour confirmed the diagnosis ABC. After 6 months, the MRI showed no recurrence. The observed tilt of the spine to the operated side on the sacral base prompted a second surgical procedure: a transpedicular fixation of L5 and L4 was connected via bent titanium stems to the ischium, where the fixation was achieved by two screws. This construction allowed the correction of the base angle and yielded a stable closure of the pelvic ring. The patient has now been followed for 6 years: the bone grafts have been incorporated and, in spite of radiological signs of screw loosening in the ischium, the patient is fully rehabilitated and free of symptoms. Pedicle screws in the lower spine can be recommended for fixation of a pelvic ring discontinuity.

**Key Words:** *aneurysmal bone cyst, pelvic reconstruction, spino ischial spondylodesils, pelvic tumor*

A 23-year-old woman presented with a low back pain (LBP) history of 2 years. Plain film radiographs and an MRI of the lumbar spine had not shown any pathologies. The patient had been treated with drugs and physical therapy. The patient was referred to our hospital since the pain increased, especially during the night with referral to the legs. A new X-ray examination of the spine showed a cystic structure in the sacral bone. Immediately following this discovery, images of the whole pelvis were taken, revealing a lytic lesion of the left pelvis involving the ilium near the acetabular roof, the sacrum near the spinal canal, and also the left L5-lamina (Fig. 1). An open biopsy was performed. The histological examination revealed multiple communicating vascular spaces separated by fibrous septa. Some areas showed suspicious bone lesions. The histological diagnosis was aneurysmal bone cyst (ABC) but, due to the very aggressive growth of the tumour, a low malignant osteosarcoma could not be excluded.

Therefore the strategy of operative treatment was an initial wide tumour resection and temporary stabilisation of the pelvic ring. After histological examination of the entire lesion, a subsequent operation was planned to reconstruct the pelvic ring with custom-made implants and graft material.

A combined anterior and posterior approach in a lateral decubitus position was used to carry out the wide tumour resection. The tumour had spread into the L5-lamina, the left part of the sacrum, the iliac crest, and the ileum down to the level of the back side of the articular cartilage of the acetabulum. Primary stabilisation of the pelvic ring was achieved by mounting a 12-hole titanium osteosynthetic plate between the L5-vertebra and the anterior inferior iliac spine (Fig. 2). Although not previously planned, the proximal part of the iliac crest, which had been removed with the tumour but was not affected by it, was dissected into two parts and used as an autologous bone graft. It was placed near the titanium plate between sacrum and hip joint and secured in place with wires.

The patient recovered without any problems. She did not suffer any peripheral neurological deficiency in her lower extremities. After the wound healing

Correspondence to: Dr. Med. Matthias Honl, Department of Orthopaedic Surgery, Eilbek General Hospital, Friedrichsberger Str. 60, 22081 Hamburg, Germany. Tel.: +49-40-2092-1344; Fax: +49-40-2092-1341; E-mail: honl@ortho-hamburg.de

1357-714X print/1369–1643 online © 2003 Taylor & Francis Ltd
DOI: 10.1080/13577140310001644805

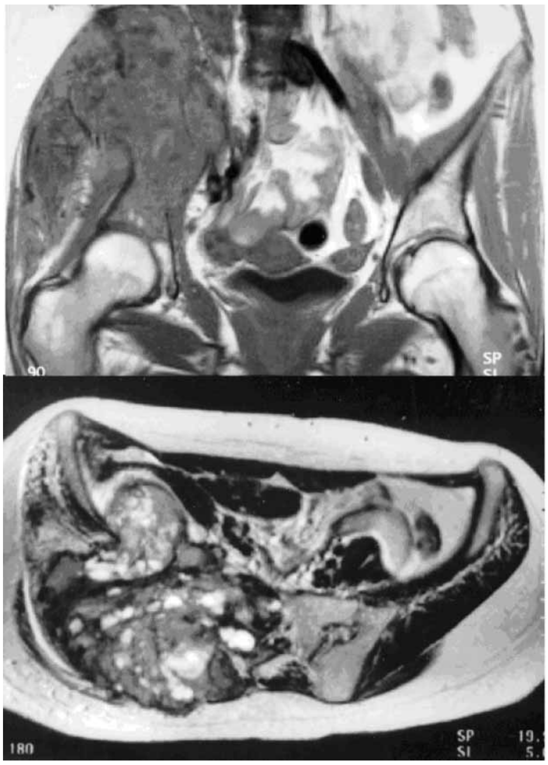

**Fig. 1.** *Tumor affecting the right pelvis: MRI. T1 and T2 sequences; T2 marks the fluid-filled spaces within the tumour.*

period, the patient was allowed to walk with the help of crutches.

The histological examination of the entire tumour confirmed the diagnosis of an aneurysmal bone cyst (Fig. 3). An MRI 6 months after the operation did not indicate any tumour recurrence. However, a tilt in the lumbar spine relative to the sacral base had occurred due to the resected muscles. Consequently the base angle of L5 to the sacral base deviated 20° from neutral to the non-operated side (Fig. 2).

For the final reconstruction, a 3-D model of the pelvis was created from CT data. Using that model, the transpedicular dorsal spondylodesis L4–S1 with a titanium transpedicular fixation system* was mounted and extended to the ischium by attaching specially designed titanium stems. These were bent to fit the 3-D model and designed to be mounted to the ischium by two pedicle screws (Fig. 4).

At the beginning of the second operation the titanium plate was removed. There was no local sign of new tumour growth. The iliac crest autologous bone graft, which had been used in the first operation, seemed to be incorporated, only the junction between the two parts of the graft showed a pseudarthrosis. The spinal and acetabular ends were well fixed. Pedicle screws were inserted into the L4- and L5-pedicles at both sides, whereas the S1-vertebra was only instrumented at the left site. Two pedicle screws were placed into the ischium: the shorter proximal one at the level of the obturator foramen, and the longer distal one anteriorly through the ischium into the pubis. A non-vascularised autologous graft of the left fibula was dissected and placed between the lumbosacral region and the ilium. Fixation was achieved using two titanium cortical screws and wire. The custom-made device was mounted at the spine and the ischium and the base angle of the spine was corrected by distraction (Fig. 5).

The early postoperative period revealed a peroneal nerve palsy due to the harvesting of the fibular graft material. Restoration of function started after about 5 weeks. For the first 6 months postoperatively, the patient was allowed weight-bearing of 50% body-weight, which she tolerated without any pain or mechanical problems. Thereafter, full weight-bearing was allowed. The nerve palsy resolved completely within 6 months.

A sequential post-operative radiograph revealed sufficient fusion without loosening zones. There was no indication of tumour recurrence. One year post-operative the patient was fully rehabilitated and returned to work. The patient has now been followed for 6 years and the overall clinical result is good. The reinforced pelvic girdle remained very stable due to the sufficient midline fixation that was achieved using the spine as the base for the mechanical construction. The most recent X-rays showed a loosening of the ischial screws with surrounding sclerosis. This was not of clinical relevance, because the pelvic ring has been re-established by the well-incorporated bone grafts. The cartilage joint space, as observed from the X-ray, and the range of motion in the hip joint is nearly normal.

### Discussion

ABC is a highly vascular bone lesion of unknown aetiology.[1] The lesion occurs by the age of 12–19 during or shortly after skeletal maturity.[1,2] It is rarely diagnosed beyond the age of 30 years.[3] It mostly arises in the long bones;[4] however, other locations are also described in the literature.[3,5–10] A few cases of aneurysmal bone cyst of the spine[11–14] or the pelvis[15,16] are reported.

For a long time the ABC was characterised as a non-neoplastic lesion.[1] More recent cytogenic studies have revealed involvement of chromosome segments which suggest that some aneurysmal bone cysts are true neoplasm.[17]

Some authors contend that an aberration of bone growth is an important factor in pathogenesis by causing alterations in haemodynamics.[18] Others describe trauma as an initial lesion for development of ABC.[8] In many cases ABC is combined with other skeletal lesions. The coexisting lesions may be

*WSI, Peter Brehm, Germany.

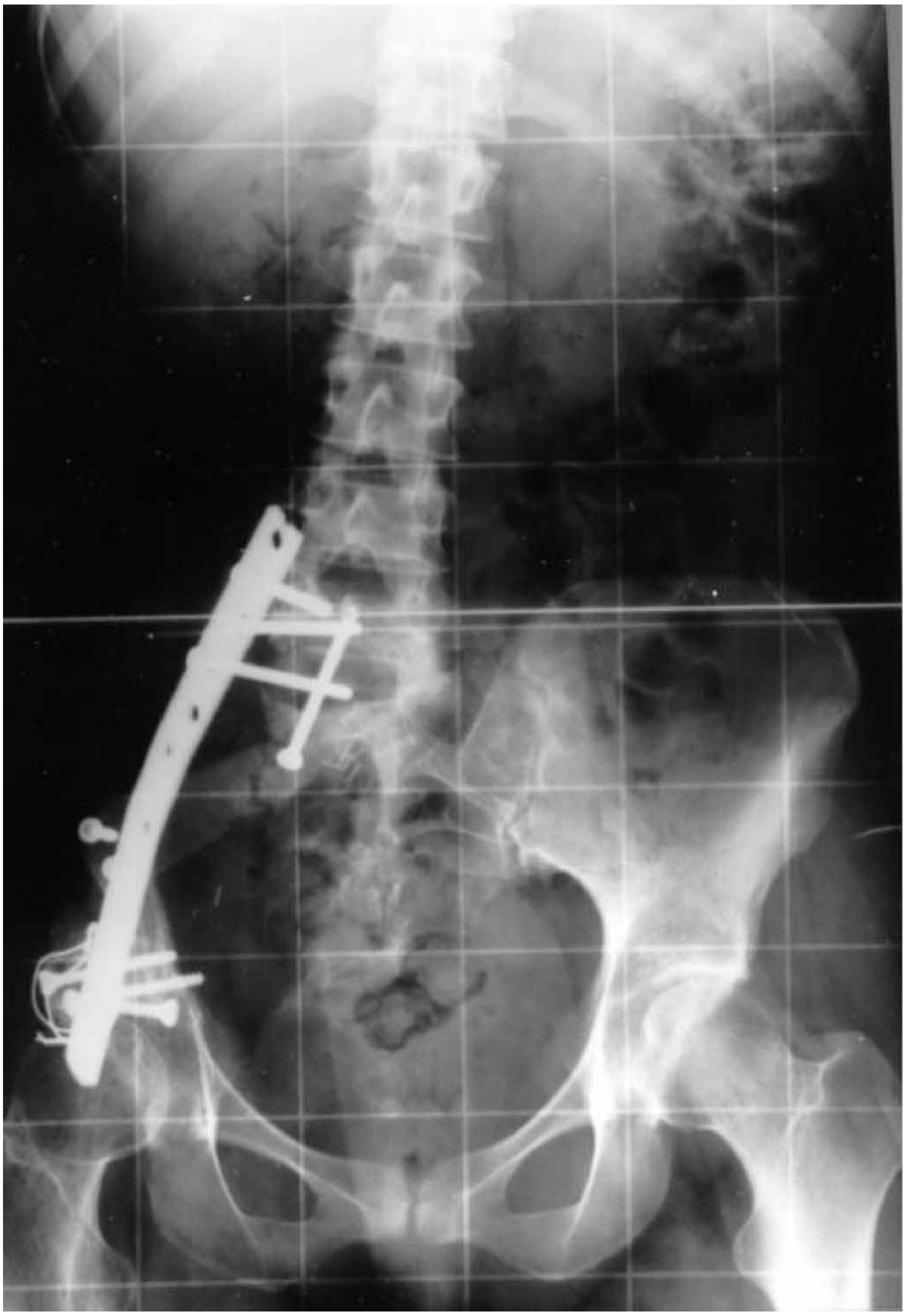

**Fig. 2.** *X-ray after tumour resection in the first operation (follow-up 6 months): a titanium plate was used for fixation. The part of the resected iliac crest, which was not affected by the tumour was used as a bone graft. Note the increasing tilt of the spine to the non-operated side.*

malignant or benign.[5,19,20] The number of coexisting lesions is estimated to be between 1 and 35%.[21]

The therapy of ABC is described as curettage, cryosurgery,[19] bone grafting or a combination of these.[22] Some authors reported selective arterial embolisation as a possible alternative or adjuvant therapy.[7,16,22–24] Radiation therapy has also been described,[25] but is not recommended due to post-radiation sarcoma. The overall management of these lesions must be individualised, depending on size, location and aggressiveness of the tumor.[16]

The rate of recurrence after curettage is reported to range from 37 to more than 50%.[19,26] Curettage and bone grafting in combination with radiation is reported with a recurrence rate of only 9%.[19] Recurrent lesions after primary surgery are noted to develop within 2 years.[16]

In this case the possibility of a low malignant osteosarcoma required the resection of the entire process. The uncertain dignity of this lesion did not allow preoperative arterial embolisation. An adjuvant chemotherapy should have been attempted if an associated osteosarcoma had been determined histologically. As, by examination of the open biopsy, there still remained doubts about the histological dignity, the strategy was a primary wide resection as

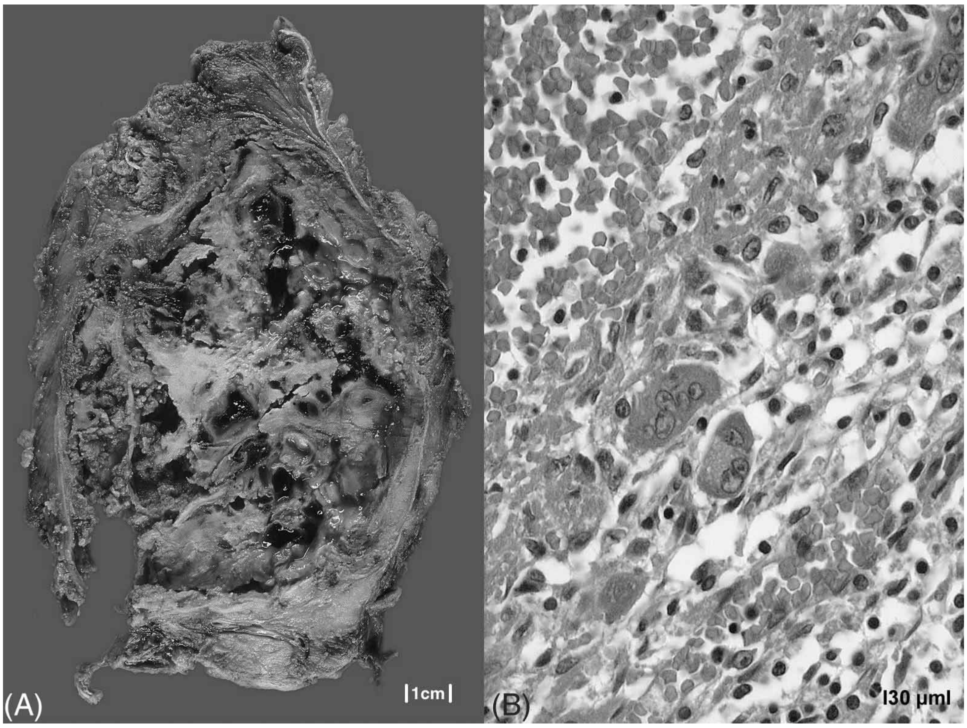

**Fig. 3.** *(A) Macroscopic aspect of the entire tumour with multiple cavities. (B) Typical histological aspect of an ABC.*

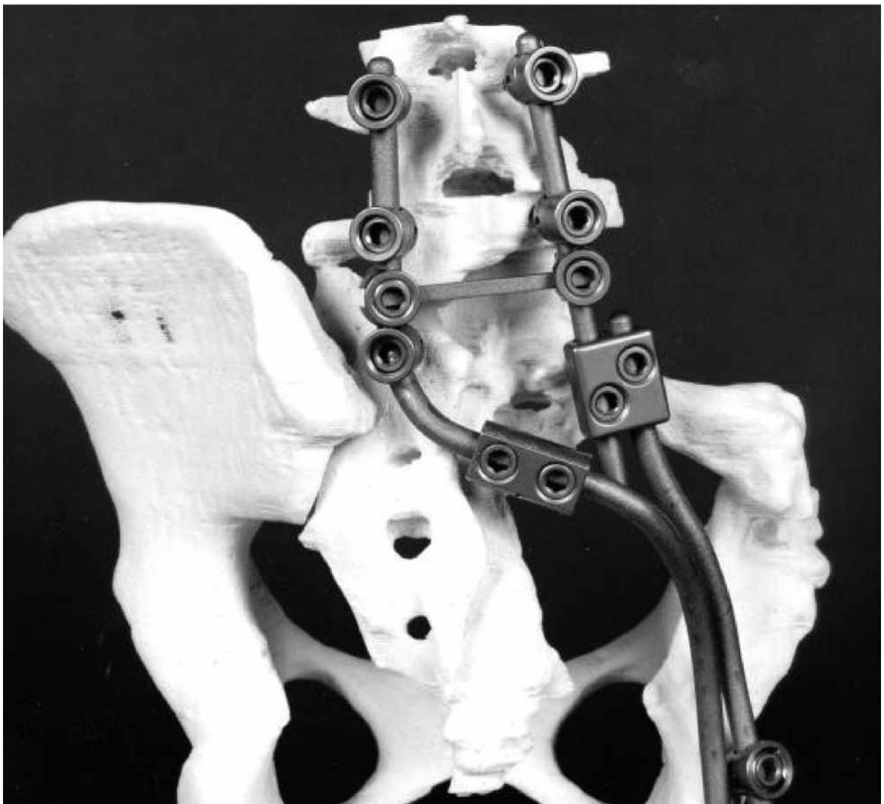

**Fig. 4.** *3-D reconstruction of the situation after the first operation: the model was used to plan the second operation. A spinal fixator was bent to the model, and two ischial screws were used to close up the pelvic ring.*

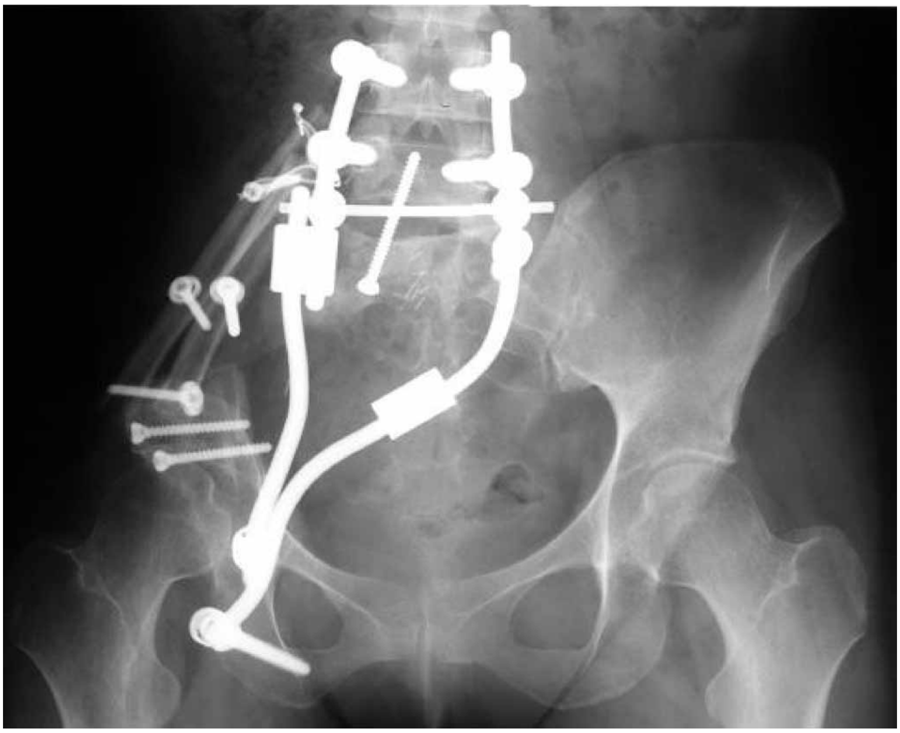

**Fig. 5.** *X-ray after the second operation. Stabilisation of the pelvic ring: two ischial screws in place, transpedicular spondylodesis and autologous bone graft; the screw visible in the midline of L5 was left after the first operation.*

recommended for large, benign, aggressive tumours undergoing malignant degeneration.[27]

The histological view of a normal aneurysmal bone cyst is composed of fibrous stroma with spindle-shaped fibroblasts, multinucleated giant cells, and inflammatory mononuclear cells. Spaces and areas of haemorrhage are uniformly present (Fig. 3). It may be difficult to distinguish this entity from a giant-cell tumour because of radiographic and histological similarities.[28] In this case the aggressive growth of the tumour meant that it was impossible to exclude a low malignant osteosarcoma by examination of the biopsy material. Low grade central osteosarcomas are very rare, they morphologically simulate benign bone tumours or tumour-like lesions (for example ABC). Because of the apparent bland histology a low malignant osteosarcoma may be misinterpreted as a benign lesion.[29] Despite their primary low malignant behaviour the capability to transform into a highly malignant sarcoma should be considered.

Limb sparing surgery in cases of pelvic tumour might be possible by the use of implants or allografts.[30–32] Pelvic reconstruction due to sacral tumour resection is technically difficult since the sacral cancellous bone does not provide sufficient mechanical stability for implant fixation. The mechanical load transfer into the pelvic ring is normally conducted via the SI-joints to the iliac bones while the sacrum itself is hardly loaded. This load transfer from the lower lumbar spine to the pelvis was achieved by the use of transpedicular and ischial screw fixation. Due to the very low bone density of the sacrum itself it is not possible to achieve implant stability by using only sacral screws. It might have been possible to load the contra-lateral SI-joint by fusing it with a trans-sacral screw. However, the more secure method seemed to be the implant fixation, as known from common lower lumbar spine fusion.

In the presented case, the tilt of the lower spine base angle favoured the use of the L5-pedicles as the base of the pelvic ring reconstruction. The tumour progression close to the acetabular roof did not allow direct implant fixation, but grafting with autologous bone was possible in this area. The pelvic ring could therefore be reconstructed with bone grafts nearly according to its normal anatomy, whereas mechanical stabilisation was achieved in a non-anatomical way.

Other authors prefer using free vascularised fibular grafts for the reconstruction of the pelvic ring.[33] In the presented case the non-vascularised grafts have been incorporated well.

This case shows a wide tumour resection and pelvis reconstruction with custom-made instrumentation and autografting. As, at a 6-year follow-up, the patient is fully rehabilitated and free of symptoms this surgical procedure can be recommended in similar cases of pelvic discontinuity due to tumour resection.

### References

1. Leithner A, Windhager R, Lang S, Haas OA, Kainberger F, Kotz R. Aneurysmal bone cyst. A population based epidemiologic study and literature review. *Clin Orthop* 1999; 363: 176–9.

2. van Loon CJ, Veth RP, Pruszczynski M, Lemmens JA, van H J. Aneurysmal bone cyst. Long-term results and functional evaluation. *Acta Orthop Belg* 1995; 61: 199–204.
3. Windhager R, Lang S, Kainberger F. Aneurysmal bone cysts. *Orthopade* 1995; 24: 57–64.
4. Yadav SS. Ankle stability after resection of the distal third of the fibula for giant-cell lesions: report of two cases. *Clin Orthop* 1981; 155: 105–7.
5. Rizzo M, Dellaero DT, Harrelson JM, Scully SP. Juxtaphyseal aneurysmal bone cysts. *Clin Orthop* 1999; 364: 205–12.
6. Ozaki T, Halm H, Hillmann A, Blasius S, Winkelmann W. Aneurysmal bone cysts of the spine. *Arch Orthop Trauma Surg* 1999; 119: 159–62.
7. Green JA, Bellemore MC, Marsden FW. Embolization in the treatment of aneurysmal bone cysts. *J Pediatr Orthop* 1997; 17: 440–3.
8. Castro MD, Irwin RB. Aneurysmal bone cyst of the patella. *Am J Orthop* 1996; 25: 717–19.
9. Konishi M, Nobuoka W. Case of aneurysmal bone cyst in sternal manubrium. *Kyobu Geka* 1996; 49: 334–6.
10. Itshayek E, Spector S, Gomori M, Segal R. Fibrous dysplasia in combination with aneurysmal bone cyst of the occipital bone and the clivus: case report and review of the literature. *Neurosurgery* 2002; 51: 815–8.
11. Stillwell WT, Fielding JW. Aneurysmal bone cyst of the cervicodorsal spine. *Clin Orthop* 1984; 187: 144–6.
12. Nicastro JF, Leatherman KD. Two-stage resection and spinal stabilization for aneurysmal bone cyst. A report of two cases. *Clin Orthop* 1983; 180: 173–8.
13. Boriani S, De IF, Campanacci L, Gasbarrini A, Bandiera S, Biagini R, *et al.* Aneurysmal bone cyst of the mobile spine: report on 41 Cases. *Spine* 2001; 26: 27–35.
14. Chan MS, Wong YC, Yuen MK, Lam D. Spinal aneurysmal bone cyst causing acute cord compression without vertebral collapse: CT and MRI findings. *Pediatr Radiol* 2002; 32: 601–4.
15. Mnaymneh W, Malinin T, Mnaymneh LG, Robinson D. Pelvic allograft. A case report with a follow-up evaluation of 5.5 years. *Clin Orthop* 1990; 255: 128–32.
16. Papagelopoulos PJ, Choudhury SN, Frassica FJ, Bond JR, Unni KK, Sim FH. Treatment of aneurysmal bone cysts of the pelvis and sacrum. *J Bone Joint Surg Am* 2001; 83-A: 1674–81.
17. Sciot R, Dorfman H, Brys P, Dal CP, De WI, Fletcher CD, *et al.* Cytogenetic-morphologic correlations in aneurysmal bone cyst, giant cell tumor of bone and combined lesions. A report from the CHAMP study group (In Process Citation). *Mod Pathol* 2000; 13: 1206–10.
18. Essadki B, Dkhissi M, Moujtahid M, Zryouil B. Aneurysmal diaphyseal bone cyst. Etio-pathogenic hypothesis and review of the literature. A case report. *Rev Chir Orthop Reparatrice Appar Mot* 1999; 85: 297–301.
19. Malawer MM, Dunham W. Cryosurgery and acrylic cementation as surgical adjuncts in the treatment of aggressive benign bone tumors. Analysis of 25 patients below the age of 21. *Clin Orthop* 1991; 263: 42–57.
20. Kransdorf MJ, Sweet DE. Aneurysmal bone cyst: concept, controversy, clinical presentation, and imaging. *AJR Am J Roentgenol* 1995; 164: 573–80.
21. Martinez V, Sissons HA. Aneurysmal bone cyst. A review of 123 cases including primary lesions and those secondary to other bone pathology. *Cancer* 1988; 61: 2291–304.
22. Athanasian EA, McCormack RR. Recurrent aneurysmal bone cyst of the proximal phalanx treated with cryosurgery: a case report. *J Hand Surg (Am)* 1999; 24: 405–12.
23. Guibaud L, Herbreteau D, Dubois J, Stempfle N, Berard J, Pracros JP, *et al.* Aneurysmal bone cysts: percutaneous embolization with an alcoholic solution of zein-series of 18 cases. *Radiology* 1998; 208: 369–73.
24. Garg NK, Carty H, Walsh HP, Dorgan JC, Bruce CE. Percutaneous Ethibloc injection in aneurysmal bone cysts. *Skeletal Radiol* 2000; 29: 211–6.
25. Marcove RC, Sheth DS, Takemoto S, Healey JH. The treatment of aneurysmal bone cyst. *Clin Orthop* 1995; 311: 157–63.
26. Ozaki T, Hillmann A, Lindner N, Winkelmann W. Cementation of primary aneurysmal bone cysts. *Clin Orthop* 1997; 337: 240–8.
27. Sar C, Eralp L. Surgical treatment of primary tumors of the sacrum. *Arch Orthop Trauma Surg* 2002; 122: 148–55.
28. Ratner V, Dorfman HD. Giant-cell reparative granuloma of the hand and foot bones. *Clin Orthop* 1990; 260: 251–8.
29. Werner M, Rieck J, Delling G. Cytogenetic changes in low grade central osteosarcomas. *Verh Dtsch Ges Pathol* 1998; 82: 189–94.
30. Kusuzaki K, Shinjo H, Kim W, Nakamura S, Murata H, Hirasawa Y. Resection hip arthroplasty for malignant pelvic tumor. Outcome in 5 patients followed more than 2 years. *Acta Orthop Scand* 1998; 69: 617–21.
31. Bruns J, Luessenhop SL, Dahmen GS. Internal hemipelvectomy and endoprosthetic pelvic replacement: long-term follow-up results. *Arch Orthop Trauma Surg* 1997; 116: 27–31.
32. Aboulafia AJ, Buch R, Mathews J, Li W, Malawer MM. Reconstruction using the saddle prosthesis following excision of primary and metastatic periacetabular tumors. *Clin Orthop* 1995; 314: 203–13.
33. Nagoya S, Usui M, Wada T, Yamashita T, Ishii S. Reconstruction and limb salvage using a free vascularised fibular graft for periacetabular malignant bone tumours. *J Bone Joint Surg Br* 2000; 82: 1121–4.
